# Intramedullary disseminated sporotrichosis in an immunocompetent patient: case report and review of the literature

**DOI:** 10.1186/s12879-023-08344-3

**Published:** 2023-07-06

**Authors:** Jennifer L. Perrault, Levi A. Endelman, Mark R. Kraemer, Derrick Chen, Wendell B. Lake, M. Shahriar Salamat

**Affiliations:** 1 University of Pikeville Kentucky College of Osteopathic Medicine, 147 Sycamore Street, Pikeville, KY 41501 USA; 2 Department of Pathology and Laboratory Medicine, University of Wisconsin, 600 Highland Avenue, Madison, WI 53792 USA; 3 Department of Neurological Surgery, University of Wisconsin, 600 Highland Avenue, Madison, WI 53792 USA

**Keywords:** Disseminated sporotrichosis, Central nervous system, Fungal infection, Intramedullary, Case report

## Abstract

**Background:**

Disseminated sporotrichosis is a severe opportunistic infection that often affects immunocompromised patients after a cutaneous inoculation. Here we present a rare case of disseminated sporotrichosis discovered as a solitary intramedullary thoracic spinal cord lesion in an immunocompetent patient.

**Case description:**

A 37-year-old man presented with progressive lower limb weakness and sensory changes over 1 week. A spinal magnetic resonance imaging (MRI) revealed a contrast-enhancing intramedullary lesion centered at T10. The patient was afebrile and reported no history of trauma or cutaneous lesions. The lesion was unresponsive to a trial of corticosteroids. A thoracic laminectomy was performed and a biopsy obtained. A cutaneous lesion on the arm was concurrently discovered, which was also biopsied. Both the skin and spinal cord biopsies showed *Sporothrix schenckii* by macroscopic and microscopic morphology which were later confirmed by MALDI-TOF mass spectrometry*.*

**Conclusion:**

This is a rare case of intramedullary disseminated sporotrichosis affecting the central nervous system of an immunocompetent patient. This unusual presentation should be taken into consideration when such intramedullary lesions are encountered.

## Background

Sporotrichosis is a fungal disease most commonly caused by a thermally dimorphic fungus, *Sporothrix schenckii,* which is a globally distributed organism. However, less commonly, sporotrichosis may also be caused by other species of the genus Sporothrix including *S. brasiliensis and S. globosa*. *Sporothrix schenckii* is primarily found in sphagnum moss, soil, hay, and decaying plants but not much is known about its ecology [1]. Sporotrichosis is typically an implantation mycosis and can present in humans in three forms: cutaneous/lymphocutaneous, pulmonary, and disseminated. Cutaneous/lymphocutaneous is the most common and presents with small sores on the affected skin which may spread along dermal and subcutaneous lymphatics. Pulmonary disease is rare and occurs when an individual, often who is immunocompromised, inhales fungal spores. Disseminated sporotrichosis most often occurs following a focal infection in individuals with immunodeficiencies and may involve the central nervous system (CNS). There are few reported cases of disseminated sporotrichosis into the CNS, which may present as isolated chronic meningitis or as a component of systemic dissemination [2]. The mechanism of *Sporothrix* dissemination to the CNS is unclear, and treatment is complicated due to treatment toxicity and difficulty sterilizing cerebrospinal fluid (CSF) in the setting of comorbid immunodeficiency [3].

## Case presentation

### Clinical presentation

A 37-year-old immunocompetent man presented with a 1 week-long progressive bilateral lower limb weakness. He had a history of tobacco use, alcohol use (5–6 drinks at a time, 2–4 times per month), and previous L3-5 posterior lumbar fusion for a traumatic L4 burst fracture 20 years earlier and no subsequent residual neurologic deficits. Initial symptoms began 2–3 weeks prior with paresthesias in bilateral feet, which he initially attributed to cold weather and the physical demands of his job as a mechanic. The patient is single and lives with a cat and reported no recent travel. Five days prior to presentation he developed rapidly progressive weakness in his bilateral lower limbs, left greater than right, and decreased sensation starting in his mid-abdomen into the bilateral legs. At presentation to a referring emergency department, he was non-ambulatory with fecal and urinary incontinence and a postvoid residual volume > 1L requiring foley catheter placement. He was transferred to our facility. Examination upon arrival demonstrated a well-developed man in no distress. Blood pressure was 126/94 mmHg, body temperature 37.6 degrees centigrade and oxygen saturation 99%. He had normal strength in his upper limbs, but severe bilateral lower limb weakness (Left: 1/5 hip flexion, 2/5 knee flexion/extension, 4/5 dorsiflexion/plantarflexion; Right 3/5 hip flexion, 4/5 knee flexion/extension, 4/5 dorsiflexion/plantarflexion), patellar and Achilles hyporeflexia, but upgoing plantar reflexes. Sensation was diminished distal to the T10 level. Pertinent laboratory findings included a white blood cell count of 5.7 K/uL (normal 3.8 – 10.5) with 67% neutrophils, C-reactive protein 0.4 mg/dL (normal 0.0 – 1.1), erythrocyte sedimentation rate 18 mm/Hr (normal 0 – 15), HIV serology and PCR negative, COVID-19 PCR negative.

**Fig. 1:**
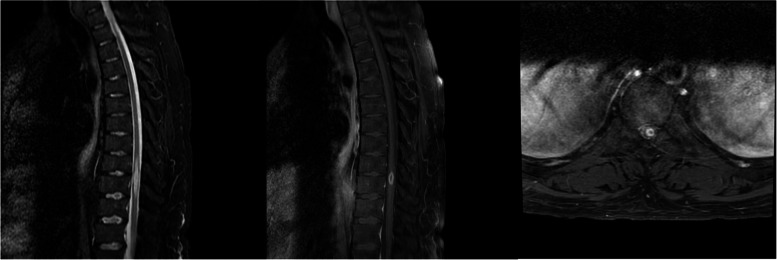
Sagittal T2 Short Tau Inversion Recovery (STIR), T1 with contrast, and axial T1 with contrast images demonstrating a 2.7 x 0.7 x 0.7 cm enhancing lesion at T10 with extensive surrounding edema extending from T6-12. There is mild enlargement of the cord at the level of the lesion

Magnetic resonance (MR) scan of the lumbar and thoracic spine demonstrated an enhancing intramedullary lesion at T10 measuring 2.7 × 0.7 x 0.07 cm with diffuse T2 cord signal extending from T6-12 (Fig. 1). Imaging of the head, cervical and lumbar spine was normal. Diagnostic considerations included transverse myelitis versus neoplasm. A lumbar puncture was performed with nucleated cell count < 1/uL (normal < 5), red cell count 1/uL (normal < 1), glucose 92 mg/dl, protein 96 mg/dl, ACE 1.8 U/L (normal 0 – 2.5), oligoclonal bands negative, gram stain and culture negative. Meningitis/encephalitis PCR panel (including *Escherichia coli*, *Haemophilus influenzae*, *Listeria monocytogenes*, *Neisseria meningitidis*, *Streptococcus agalactiae*, *Streptococcus pneumoniae*, CMV, enterovirus, parechovirus, VZV, *Cryptococcus neoformans*, and HSV) was negative. Flow cytometry was negative for malignancy.

His strength declined over the course of the first several days after admission with near complete paraplegia of the lower limbs. He was started on high-dose IV methylprednisolone (1000 mg) IV every day for 5 days with significant improvement to antigravity strength at the knee and ankle. Repeat MRI performed after one week demonstrated decreased size of the enhancing lesion (from 2.7 × 0.7 × 0.7 cm to 1.7 × 0.7 × 0.7 cm) and decreased surrounding edema.

After completing the initial course of corticosteroids, lower limb strength again declined 4 days later prompting repeat MR imaging demonstrating recurrent extensive T2 cord signal change, similar to the initial scan. The patient’s case was discussed at the institutional multi-disciplinary spine/neuro-oncology conference where inflammatory etiologies were considered most likely. The decision was made to continue corticosteroids, initiate plasmapheresis and perform a 1-month follow-up MRI prior to consideration of spinal cord biopsy.

He underwent five plasmapheresis treatments and a second course of high-dose IV methylprednisolone (1000 mg every other day × 5, alternating with plasmapheresis treatments) and subsequently was placed on an oral prednisone taper (60 mg by mouth (PO) daily (QD) for 26 days followed by 50 mg PO QD for two weeks, 40 mg PO QD for two weeks, 30 mg PO QD for two weeks, and has been on 20 mg PO QD indefinitely as maintenance treatment). Lower limb strength waxed and waned. Repeat imaging (brain and total spine) was again performed as planned approximately 1 month after admission with stable appearance of the thoracic lesion, but progression of T2 cord signal. Repeat CSF sampling showed a similar profile to earlier sampling. Given the stable appearance of the lesion and persistent deficits, spinal cord biopsy was offered. A T9-10 laminectomy was performed with microsurgical partial resection of the lesion using SSEP and MEP monitoring.

### Pathology and Microbiology

**Fig. 2:**
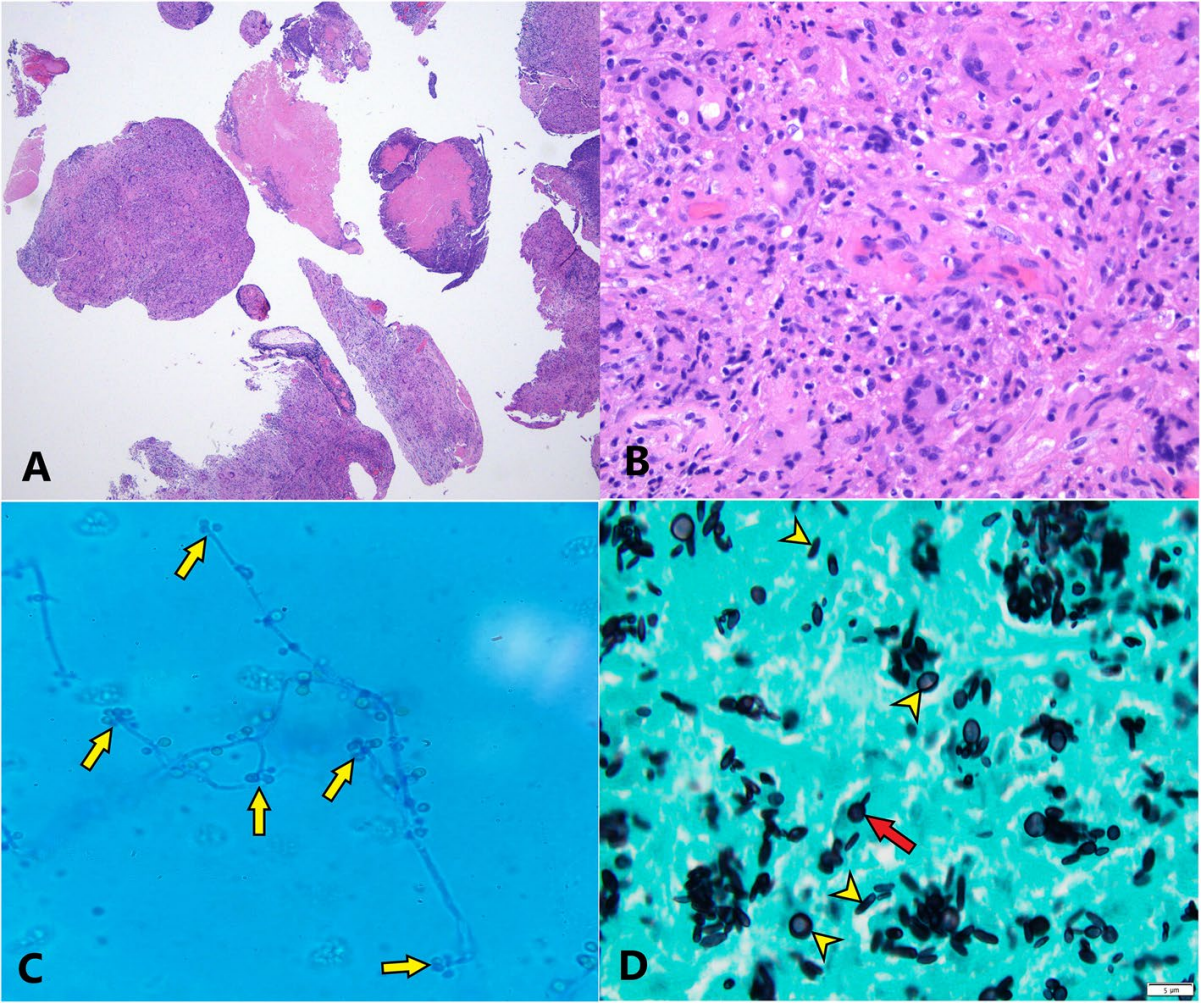
**A** Intramedullary spinal cord lesion, 4 × objective magnification H&E, revealing areas of granulation tissue and necrosis. **B** Intramedullary spinal cord lesion, 40 × objective magnification H&E, revealing granulomatous inflammation, necrosis, and multinucleated giant cells. **C** Intramedullary spinal cord lesion fungal colony, 100 × objective magnification with oil immersion of lactophenol blue tape preparation, revealing hyphal morphology with microconidia budding along the length of hyphae with rosettes of microconidia at the terminal ends of hyphae (yellow arrows). **D** Intramedullary spinal cord lesion, 100 × objective magnification with oil immersion of GMS stain, revealing individual yeast forms measuring 2–6 µm in length and demonstrating spherical and characteristic “cigar-shaped” morphology (yellow arrowheads) with occasional cigar-shaped buds arising from spherical forms (red arrow)

**Fig. 3:**
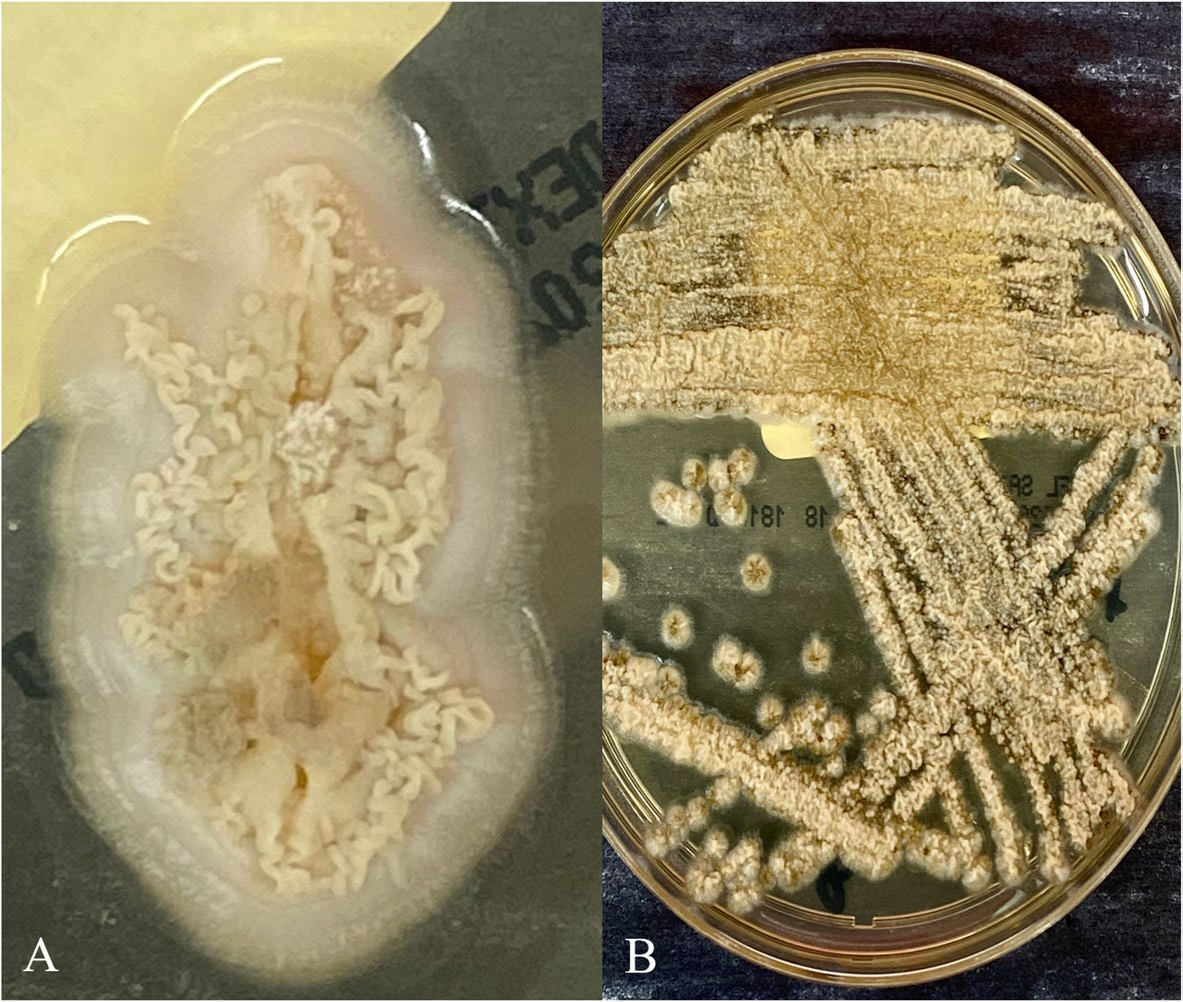
Fungal colony photographs. **A** Closeup image of an individual fungal colony from spinal cord specimen revealing characteristic finely wrinkled colony morphology (potato dextrose agar). **B** Fungal streak plate from spinal cord specimen (Sabouraud dextrose agar)

**Fig. 4:**
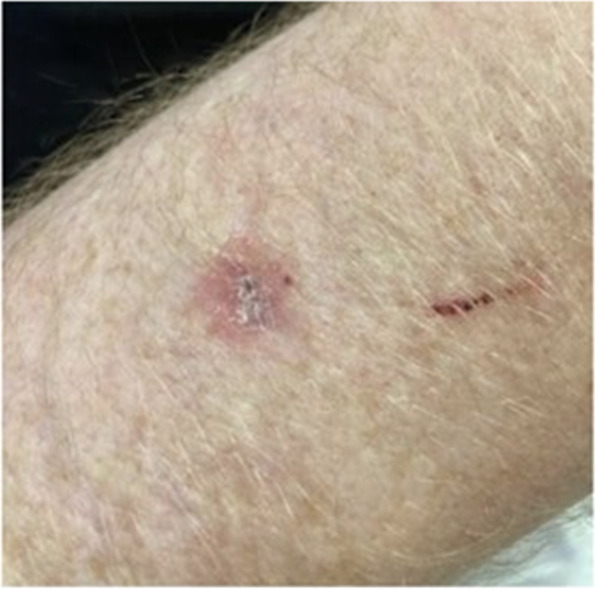
Dorsal view, left forearm revealing a 1.1 cm firm red nodule with central area of necrosis

**Fig. 5:**
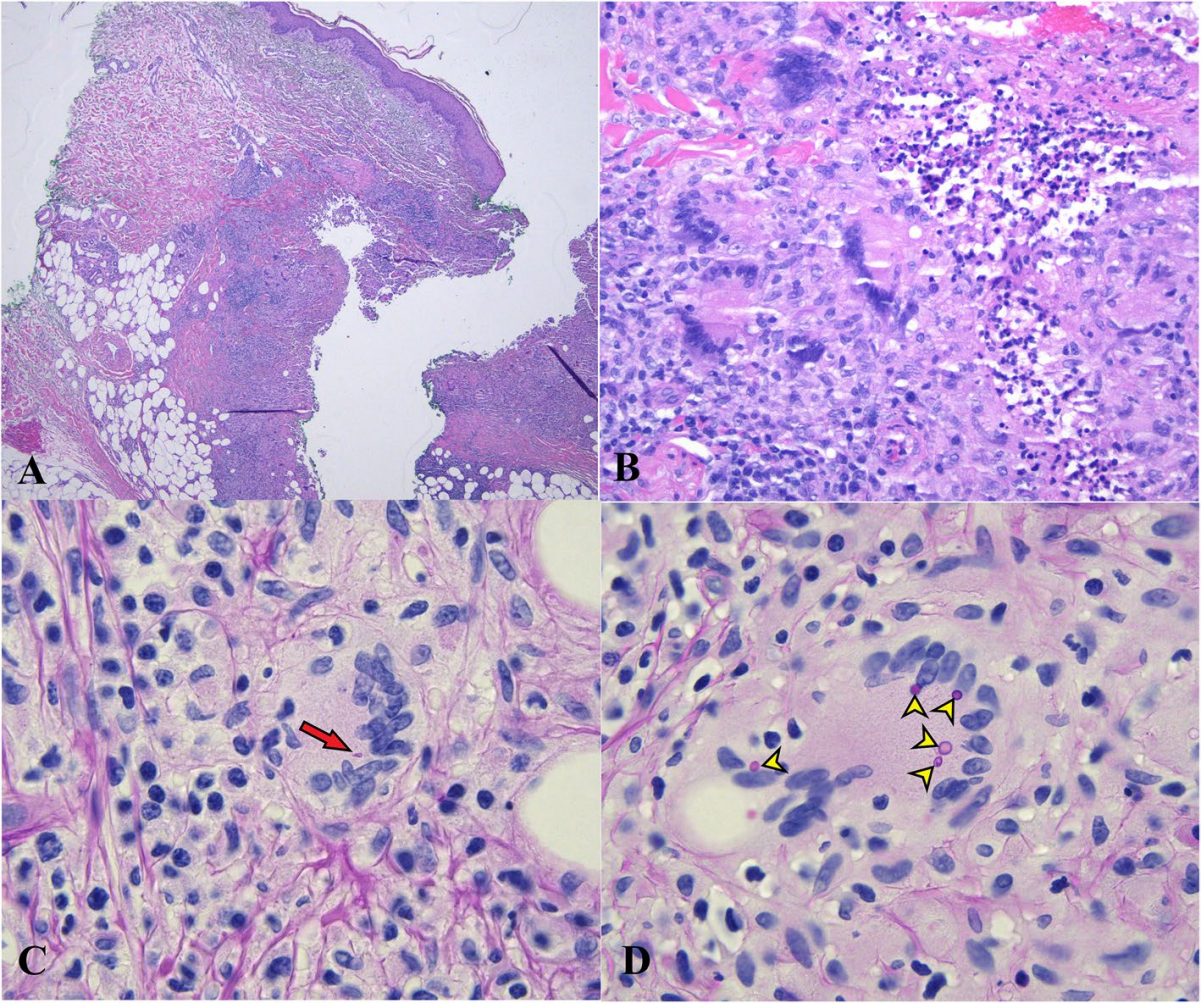
**A** Left upper arm lesion, 4 × objective magnification H&E, revealing skin with a dense inflammatory infiltrate extending into the deep dermis and subcutaneous adipose tissue. **B** Left upper arm lesion, 40 × objective magnification H&E, revealing granulomatous inflammation, multinucleated giant cells, and areas of necrosis. **C** Left upper arm lesion, 100 × objective magnification with oil immersion of PAS stain, revealing a rare PAS positive cigar-shaped organism (red arrow) within a multinucleated giant cell. **D** Left upper arm lesion, 100 × objective magnification with oil immersion of PAS stain, revealing multiple PAS positive spherical organisms (yellow arrowheads) within a multinucleated giant cell

Histologic and microbiologic analysis of the spinal cord lesion including examination of both macroscopic and microscopic fungal culture morphology as well as MALDI-TOF mass spectrometry revealed the presence of *Sporothrix schenckii* (Figs. 2 and 3)[4, 5]. Given these initial findings, infectious disease was consulted and empiric IV Amphotericin B was initiated. The infectious disease team additionally noted a lesion on the patient’s left arm (Fig. 4). This was not observed upon admission, nor mentioned by the patient. Dermatology performed a punch biopsy of the 1.1 cm firm red nodule. Cultures and MALDI-TOF mass spectrometry of this lesion also recovered and identified *Sporothrix schenckii*. Histology of this lesion revealed extensive granulomatous inflammation in the dermis, extending into the subcutaneous adipose tissue along with several multinucleated giant cells and areas of necrosis (Fig. 5A, B). Both cigar-shaped and spherical PAS positive organisms were identified within the multinucleated giant cells (Fig. 5C, D). He was subsequently treated with a two-week course of amphotericin B followed by oral itraconazole. He was discharged to rehabilitation and remains wheelchair bound.

## Discussion

This is a rare case of disseminated sporotrichosis infection with involvement of the thoracic spinal cord, which to our knowledge is the first published report of this kind. The majority of disseminated sporotrichosis occurs in immunocompromised patients, particularly those with HIV infection [6]. Our patient was immunocompetent, but regularly consumed alcohol, tobacco, and had had prior surgical manipulation with orthopedic hardware placed near the affected site, all potential risk factors. Douraiswami et al. reported a case (Table 1) in which an immunocompetent patient with disseminated sporotrichosis presented with upper back pain and numbness in the L1 dermatome. Imaging demonstrated an extradural mass at T12-L2, which tested positive for *S. schenckii* [7]*.* This case is similar to our patient in that he did not have any history of trauma, fever, or obvious cutaneous lesions. However, our patient’s infection was found in the extradural space.

**Table 1:** Previously published cases of disseminated sporotrichosis into the CNS

Author, country, year, reference	Age (years), sex	Initial symptoms	Underlying condition	*Sporothrix* species	Location of infection	Treatment	Outcome
Douraiswami, India, 2019, [7]	35, M	Upper back pain and numbness of anterior right thigh	None	*S. schenckii*	Extradural mass on spinal cord	Amphotericin B and Itraconazole	Recovered
Paixão, Brazil, 2015, [8]	20, F	Cutaneous lesions that began on left hand and spread	HIV	*S. brasiliensis*	Skin (initial), blood, CSF, sputum, urine	Amphotericin B and Itraconazole	Died (undetermined cause),
Vilela, Brazil, 2007, [9]	34, M	Vomiting, nausea, fever, oral candidiasis, cutaneous lesions throughout body	HIV	*S. schenckii*	Skin and CSF	Amphotericin B	Died (undetermined cause)
Mialski, Brazil, 2018, [10]	46, M; 40, M	46, M: headaches, retro-orbital pain, confusion, gait impairment 40, M: weight loss, lethargy, headaches, vomiting, confusion	46, M: None 40, M: None	46, M: *S. brasiliensis* 40, M: *S. brasiliensis*	46, M: CSF 40, M: CSF	46, M: Amphotericin B and Fluconazole 40, M: Amphotericin B	46, M: Good clinical response, no relapses 40, M: Recurrent episodes of hydrocephalus, still hospitalized

*Sporothrix schenckii* is a thermally dimorphic fungus found worldwide, primarily in soil and on decaying plant matter, and is the cause of sporotrichosis, also referred to as “rose handler’s disease.” [11] At room temperature (25 °C) in the environment or when grown in laboratory conditions it assumes its hyphal form [12] which consists of thin, delicate, septate hyphae measuring 1–2 µm in diameter [13]. Small microconidia of approximately 3–6 µm diameter develop along the length of the hyphae, connected to an individual hypha by thin, hairlike projections [13]. Microconidia develop in clusters and rosettes at the terminal ends of conidiophores (Fig. 2C) [13]. Colonies grown in culture demonstrate white to yellow coloration with a wrinkled to folded surface appearance (Fig. 3A-B) [13]. Darker gray to black pigmentation can develop in mature colonies due to production of melanin and melanin-like pigments [12, 13].

At body temperature (37 °C) it assumes its yeast form with individual organisms measuring 2–4 µm in greatest dimension with spherical to oval morphology [13]. A second, cigar-shaped morphology is also seen with individual organisms measuring 2–6 µm in length and demonstrating an elongated, slender shape with blunted ends. Cigar-shaped buds can occasionally be seen budding off of larger spherical yeast forms (Fig. 2D) [13]. Tissue reaction to *Sporothrix schenckii* includes geographic necrosis, formation of granulation tissue, mixed inflammation, and the presence of multinucleated giant cells (Fig. 2A-B). “Asteroid bodies” may also be seen, however they are not present in this case.

Sporotrichosis has a high prevalence of zoonotic transmission, especially via cats [1], a pertinent risk factor in this case. Another case of CNS involvement of sporotrichosis began with a cat scratch which led to an ulcerated lesion on the patient’s hand [8]. The fungus was found in the patient’s cerebrospinal fluid (CSF) and she developed hydrocephalus resulting in headache, nystagmus, and confusion [8]. In this case (Table 1), the CSF culture grew *S. brasiliensis*, an especially virulent species of *Sporothrix* found in Brazil.

While our patient had CSF cytology performed, it did not raise suspicion for infection and CSF bacterial cultures, not fungal cultures, were initially performed on the available CSF specimen. In the case reported by Paixão et al., the patient’s CSF was cultured using Sabouraud, Mycosel, and BHI-Agar [8]. If we had cultured our patient’s CSF to look for fungal growth, a diagnosis may have been made sooner. However, in a study consisting of 48 patients with HIV plus disseminated sporotrichosis, only 3 patients had fungal growth in the CSF [3]. Kauffman also states that *Sporothrix* does not usually grow in the CSF of patients who have isolated chronic meningitis as a result of sporotrichosis, but it will grow in patients with HIV and disseminated sporotrichosis [2].

Due to the lack of a previously known cutaneous lesion and a fever, we elected to perform a spinal cord biopsy for diagnosis. Spinal cord biopsy is a high-risk procedure and remains a diagnostic tool of last resort. Dormegny et al*.* have outlined appropriate indications for this diagnostic study. If a diagnosis cannot be made after imaging, physical examination, and/or blood/CSF studies, further investigation is necessary which includes biopsy of other available organs, such as a skin lesion [14]. If a diagnosis is still not made and the patient is progressively worsening, a spinal cord biopsy may be considered.

## Conclusion

This case illustrates diagnosis of disseminated sporotrichosis after biopsy of a thoracic intramedullary lesion in an immunocompetent patient with no reported history of trauma, fever, and no initially reported cutaneous lesions. In our patient, testing the CSF specifically for fungal growth and performing a thorough skin exam may have resulted in quicker diagnosis and avoidance of a spinal cord biopsy.

## Data Availability

The datasets used and/or analyzed during the current study are available from the corresponding author on reasonable request.

## Abbreviations

CMV: Cytomegalovirus
CNS: Central nervous system
CSF: Cerebrospinal fluid
MRI: Magnetic resonance image
HIV: Human immunodeficiency virus
HSV: Herpes simplex virus
IV: Intravenous
H&E: Hematoxylin and eosin
GMS: Gomori methenamine silver
MALDI-TOF: Matrix-assisted laser desorption/ionization-time of flight
VZV: Varicella zoster virus
